# High dropout rate from aftercare program of antihepatitis C therapy for patients with history of injection drug use

**DOI:** 10.1002/jgh3.12376

**Published:** 2020-06-23

**Authors:** Akihiro Tamori, Sawako Uchida‐Kobayashi, Ritsuzo Kozuka, Hiroyuki Motoyama, Kanako Yoshida, Naoshi Odagiri, Kohei Kotani, Etsushi Kawamura, Hideki Fujii, Atsushi Hagihara, Masaru Enomoto, Norifumi Kawada

**Affiliations:** ^1^ Department of Hepatology, Graduate School of Medicine Osaka City University Osaka Japan; ^2^ Department of Premier Preventive Medicine, Graduate School of Medicine Osaka City University Osaka Japan

**Keywords:** direct‐acting antivirals, dropout, hepatitis C virus, injection drug use, person who injecting drugs

## Abstract

**Background and Aim:**

We assessed direct‐acting antiviral (DAA) treatment for patients with hepatitis C virus (HCV) and a history of injection drug use (IDU) in Japan.

**Method:**

This retrospective observational study was based on clinical records. Overall, 804 DAA‐naïve HCV‐infected patients were enrolled, treated with a 12‐week regimen of DAAs, and had available information about a history of IDU. Anti‐HCV efficacy was defined as a sustained viral response 12 weeks post‐treatment (SVR12) only in patients who were assessed after 12 weeks [modified intention‐to‐treat (ITT) analyses]. We compared the antiviral effect between patients with (past‐IDU) and without a history of IDU (non‐IDU). We also evaluated the characteristics of each group, including the overall dropout rate and economic background.

**Results:**

Overall, 78 (9.7%) patients had a history of IDU. Compared to the non‐IDU group at baseline, the past‐IDU group consisted of predominantly male and younger patients infected with HCV genotype 2. Overall, 3% (3/78) and 16% (116/726) of the patients had cirrhosis in the past‐IDU and non‐IDU group, respectively. There was a significantly higher rate of welfare recipients in the past‐IDU group. SVR rate was 97% (59/61) in the past‐IDU group and 99% (689/699) in the non‐IDU group. The cumulative rate of dropout from an aftercare program was high in the past‐IDU group (*P* < 0.01).

**Conclusions:**

DAAs had a remarkable anti‐HCV effect in patients with past‐IDU who continued in an aftercare program. It is necessary to understand the characteristics of past‐IDU patients to establish a support system for aftercare programs.

## Graphical abstract

The cumulative dropout rates from the aftercare program of antihepatitis C therapy were significantly higher in the past‐IDU group compared to those in the non‐IDU group (*P* < .01).

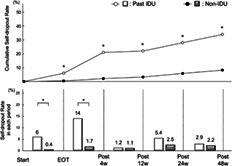

## Introduction

Globally, it is estimated that 71.1 million individuals are chronically infected with hepatitis C virus (HCV), of whom 10–20% will develop liver complications including decompensated cirrhosis and hepatocellular carcinoma (HCC).^1^ The recently approved direct‐acting antivirals (DAAs) can achieve a >95% sustained viral response (SVR) rate for DAA‐naïve patients with HCV.^1^ In Japan, all HCV‐infected patients can receive DAA therapy via a public support system for viral hepatitis,^2^ including decompensated cirrhotic patients. In addition, aftercare programs are recommended for SVR patients to detect the development of HCC earlier and to evaluate improvement of liver function. Japan aims to eliminate HCV by the end of 2030, as defined in the World Health Organization (WHO) strategy.^3^, ^4^

Recently, several countries have focused on people who inject drugs (PWID) as an important strategy to help eliminate HCV.^5^, ^6^ Drug use is the primary transmission route of HCV, and reinfection is common in PWID and in prisoners after DAA therapy.^7^ Use of a needle/syringe program combined with opioid substitution therapy can reduce HCV acquisition in PWID.^8^

However, there are few reports about HCV reinfection and HCV prevalence in PWID in Japan.^9^, ^10^ Illicit injection drugs are illegal, and it is difficult for general clinicians to initiate DAA therapy for patients who currently use injected drugs in Japan. Hence, in this study, we retrospectively identified patients with a history of injection drug use (IDU) who were treated with DAAs. We compared the clinical characteristics at baseline and the efficacy of DAA treatment between patients with past IDU and patients without IDU.

### *Patients and methods*

#### *Cohort*

We used the clinical records of HCV‐infected patients who were treated with interferon‐free DAAs between September 2015 and September 2019 at Osaka City University Hospital.

#### *Study design and* *HCV* *therapy*

**Figure 1:**
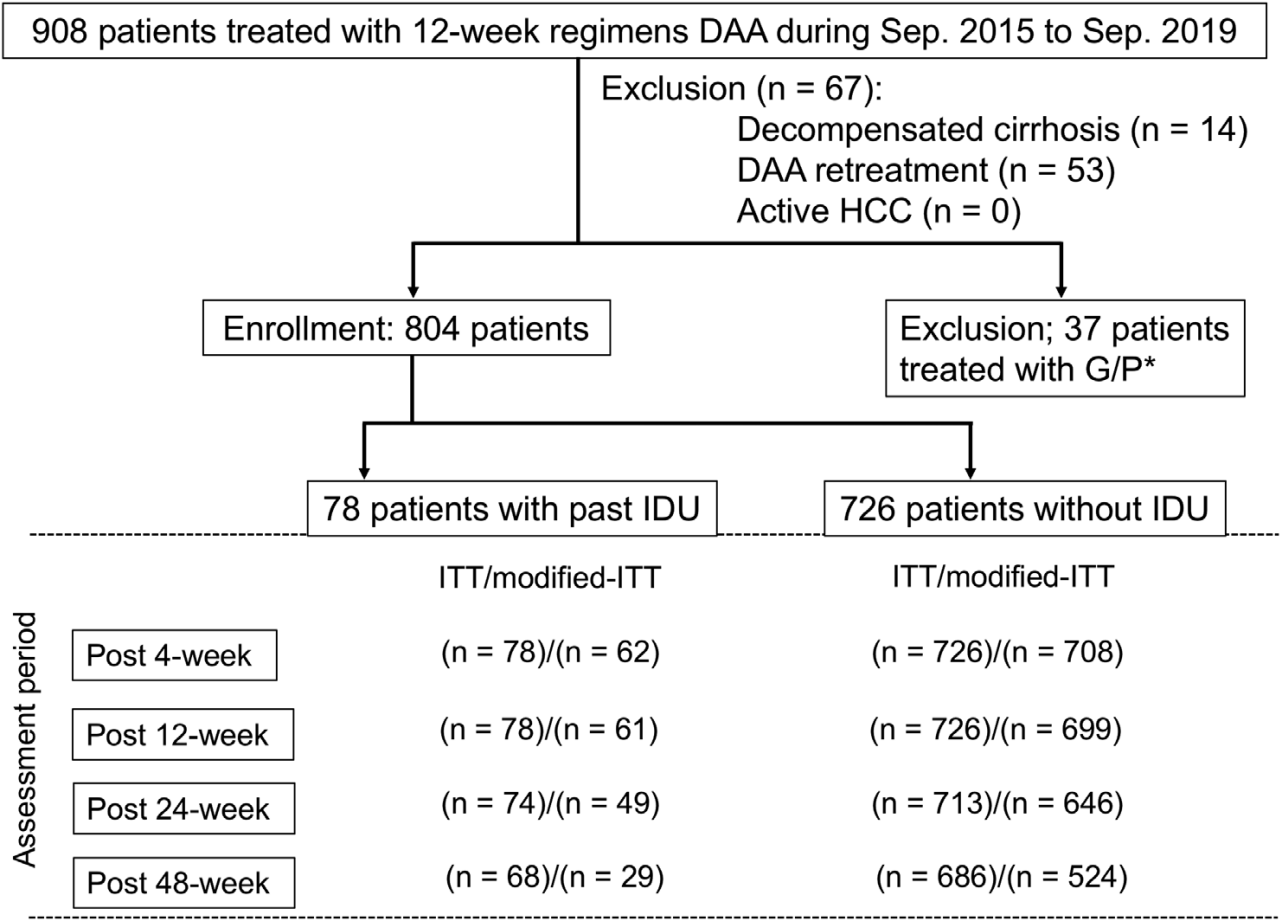
Flow diagram of the present study. According to inclusion criteria, 804 patients were enrolled the study; 37 patients treated with glecaprevir and pibrentasvir were excluded because these patients were evaluated in a previous report (15). Twelve weeks passed from the end of the direct‐acting antiviral (DAA) treatment in all enrolled patients. In addition, 48 weeks passed in 68 patients with past injection drug use (IDU) and 686 patients without IDU.

Overall, 804 patients were enrolled in this retrospective observational study. There were 408 females and 396 males, with a median age of 68 years old (21–90 years) (Fig. 1). Inclusion criteria included being DAA‐naïve, having undergone a 12‐week regimen of DAAs, noncirrhotic or compensated cirrhotic patients with chronic HCV infection, and having available information about history of IDU. The one exclusion criterion was having noncurative HCC. Overall, 27 patients were treated with 25 mg ombitasvir (OBP) + 150 mg paritaprevir (PTV) + 100 mg ritonavir per day, 104 patients were treated with 100 mg grazoprevir (GZR) and 50 mg elbasvir (EBR) per day, 446 patients were treated with 400 mg sofosbuvir (SOF) and 90 mg ledipasvir (LDV) per day, and 230 patients were treated with 400 mg SOF and bodyweight‐arranged dose of ribavirin (RBV) per day, in accordance with guidelines from the Japanese Society of Hepatology^11^ (Table 1). During the follow‐up period, clinical, biochemical, and quantitative serum HCV RNA assessments were evaluated at 1–3‐month intervals. Liver cirrhosis was defined as stage F4 based on histological examinations and according to the METAVIR scoring system or based on a transient elastography of >14.6 kPa combined with a platelet count <10 × 104/μL and the presence of cirrhotic features in ultrasonography (e.g., with respect to liver size, nodularity, caudate hypertrophy, echogenicity, and spleen size). We compared the antiviral effects of each DAA between groups. We also evaluated the characteristics of each group, including the overall dropout rate and economic background. This study protocol was approved by the Ethics Committee of Osaka City University Hospital (No. 3131, 3212, 3439, and 3619). We have provided enrolled patients the opportunity to opt out in our home page (http://www.med.osaka-cu.ac.jp/liver/).

**Table 1:** Baseline characteristics of patients with past injection drug use (IDU) and patients without IDU

	Past‐IDU (*n* = 78)	Non‐IDU (*n* = 726)	*P*‐value
Age	53 (31–84)	68 (21–90)	<0.01
number; aged ≥ 70 years	9	314	<0.01
Gender (male/female)	62/16	334/392	<0.01
Cirrhosis +/−	3/75	116/610	<0.01
Past HCC +/−	2/76	89/637	<0.01
Past IFN therapy +/−	16/76	193/529	0.26
Hemodialysis +/−	0/78	13/713	0.23
BMI 25≤/25>	34/44	191/531	<0.01
BMI 30≤/30>	7/71	33/689	0.09
Welfare recipients/nonwelfare recipients	49/29	144/582	<0.01
HCV genotype 1/2	20/58	504/222	<0.01
HCV RNA (log IU/mL)	6.45 (1.4–7.6)	6.2 (2.1–7.7)	0.12
NS5A; RAS^†^ in L31 and/or M93/Wild in both L31 and M93/ND	1/19/58	67/424/235	0.29
HBsAg +/−/ND	1/76/1	10/706/10	0.94
Anti‐HBc +/−/ND	29/47/2	257/440/29	0.82
Plt (×10^4^/μL)	19.5 (6.6–34.2)	16.7 (1.3–43.8)	<0.01
Alb (g/dL)	4.2 (3.2–4.9)	4.0 (2.4–5.0)	<0.01
T Bil (mg/dL)	0.5 (0.1–1.3)	0.6 (0.1–2.7)	0.05
ALT (U/L)	40 (10–320)	36 (8–610)	0.06
g‐GTP (U/L)	48 (9–634)	33 (2–707)	<0.01
eGFR (30 mL/ min /1.73 m^2^)	77.1 (45.8–141.9)	73.7 (0.5–166.5)	0.02
FIB‐4 index^†^	1.80 (0.49–4.78)	2.67 (0.33–49.75)	<0.01
DAA regimens			
PTVr + OBV	1	26	
SOF + RBV	34	196	
SOF + LDV	35	410	
GZR + EBR	8	94	

† The fibrosis‐4 index was calculated using Sterling's formula: age (years) × AST (U/L)/platelet count (×10^9^/L) × √ALT (U/L).

EBR, elbasvir; eGFR, estimated glomerular filtration rate; GZR, grazoprevir; LDV, ledipasvir; OBV, Ombitasvir; PTVr, Paritaprevir + Ritonavir; RAS, resistance‐associated substitution; RBV, ribavirin; SOF, sofosbuvir.

#### *Assessment of* *HCV* *and dropouts*

SVR was defined as undetectable HCV RNA in the serum at 12 weeks post‐treatment. SVR was only assessed in the modified ITT population, which excluded patients who dropped out before HCV RNA assessment at 12 weeks. HCV RNA was examined until 48 weeks after treatment in SVR patients. All methods of assessing treatment effectiveness were in accordance with existing guidelines.^12^ HCV RNA was determined using the TaqMan HCV assay (COBAS TaqMan HCV assay; Roche Molecular Diagnostics, Tokyo, Japan) with a lower limit of quantification of 15 IU/mL and an upper limit of 6.9 × 107 IU/mL (range: 1.2–7.8 log IU/mL). HCV genotype (GT) was determined using an HCV genotype primer kit (Institute of Immunology Co., Ltd., Tokyo, Japan).

From treatment initiation to 48 weeks post‐treatment, dropout rate was evaluated at end of treatment (EOT) and after 4, 12, 24, and 48 weeks. Dropout was defined as patients who stopped care by themselves.

### *Statistical analyses*

All statistical analyses were conducted using JMP software (ver. 12.0; SAS Institute, Cary, NC, USA). Continuous variables were compared between groups using the Mann–Whitney *U* test, and discontinuous variables were compared using the *χ* ^2^ test or Fisher's exact test. A *P*‐value < 0.05 was considered significant for two‐tailed tests.

## Results

**Figure 2:**
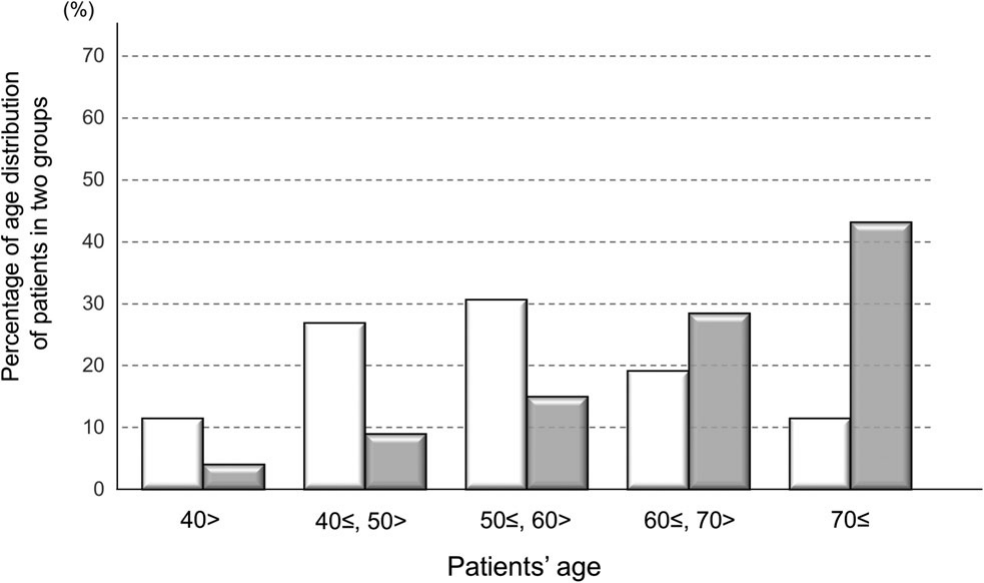
Percentage of age distribution of patients in the past‐IDU (injection drug use) and non‐IDU groups. In age distribution, the 50s age group was predominant in the past‐IDU group. The number of patients increased at more advanced ages in the non‐IDU group. 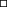, Past IDU; 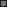, Non‐IDU

Clinical records at baseline showed that 78 (9.7%) patients had a history of IDU, and these patients were significantly younger and predominantly male (Table 1). In age distribution, the 50s age group was predominant in the past‐IDU group (Fig. 2). On the other hand, the number of patients increased at more advanced ages in the non‐IDU group. Overall, 3% (3/78) and 16% (116/726) of the patients had cirrhosis in the past‐IDU and non‐IDU groups, respectively (*P* < 0.01). The values were 3% (2/76) and 12% (89/726) for curative HCC (*P* < 0.01) and 74% and 31% for HCV GT2 (*P* < 0.01), respectively. In addition, there were significantly more welfare recipients (63%) in the past‐IDU group (*P* < 0.01).

**Figure 3:**
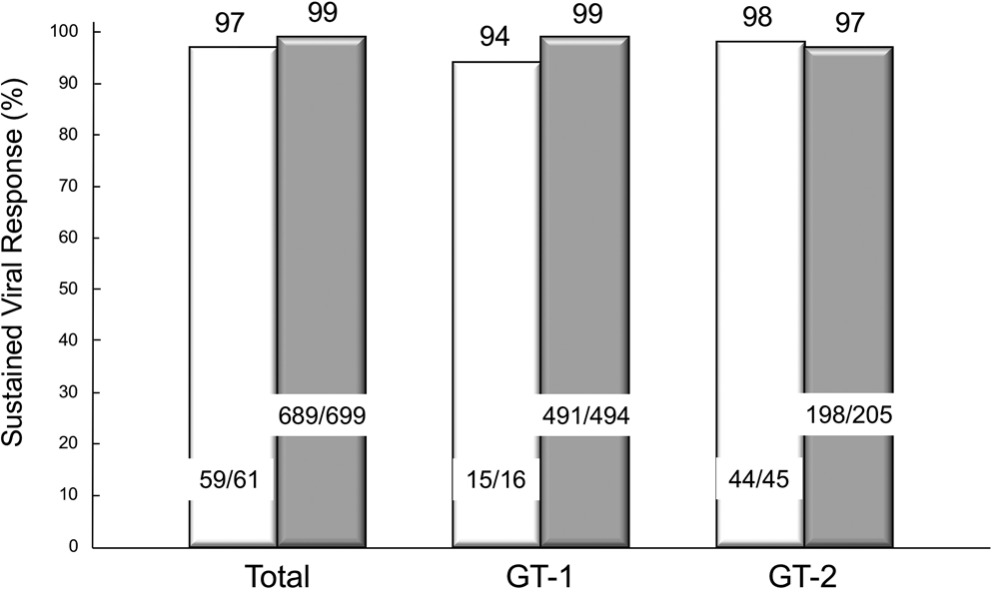
Sustained viral response in modified ITT analyses. The sustained viral response (SVR) rate was 97% (59/61) in the past‐IDU (injection drug use) group and 99% (689/699) in the non‐IDU group. Among GT1 patients, it was 94% in the past‐IDU group and 99% in the non‐IDU group, and among GT2 patients, it was 97% in the past‐IDU group and 99% in the non‐IDU group. There were no statistically significant differences in the SVR rate between the two groups. 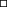, Past IDU; 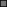, Non‐IDU

**Figure 4:**
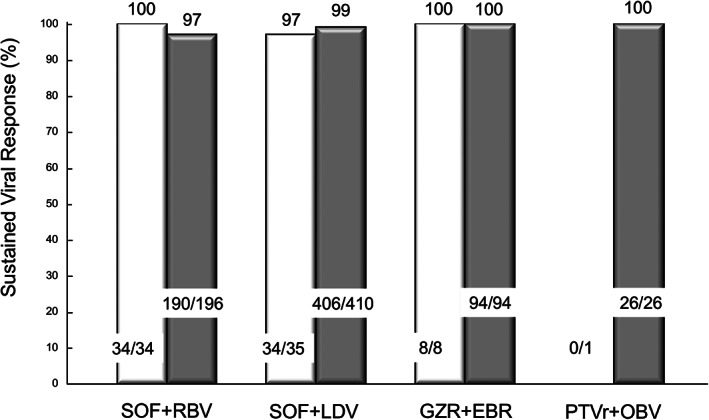
Sustained viral response in modified ITT analyses by each direct‐acting antiviral (DAA) regimen. sustained viral response (SVR) rate was not statistically significant between patients with and without a history of injection drug use (IDU) in each DAA regimen. 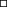, Past IDU; 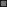, Non‐IDU

SVR was achieved in 97% (59/61) and 99% (689/699) of past‐IDU and non‐IDU patients, respectively (*P* = 0.57). Among GT1 patients, 15 of 16 (94%) patients with past IDU achieved an SVR, and 491 of 494 (99%) patients without IDU achieved it (*P* = 0.28). Among 250 patients with GT2, the SVR rate was 98% (44/45) in the past‐IDU group and 97% (198/205) in the non‐IDU group (Fig. 3). Among patients treated with SOF + RBV, the SVR rate was 100% (34/34) in the past‐IDU group and 97% (190/196) in the non‐IDU group (Fig. 4). Among patients treated with SOF + LDV, the SVR rate was 97% (34/35) in the past‐IDU group and 99% (406/410) in the non‐IDU group. Among patients treated with GZR + EBR, the SVR rate was 100% in both the past‐IDU group and the non‐IDU group. After SVR assessment, HCV RNA was redetected in two non‐IDU patients. HCV reinfection was suspected in one of them, as previously reported.^8^ There were no patients with suspected reinfection in the past‐IDU group.

**Figure 5:**
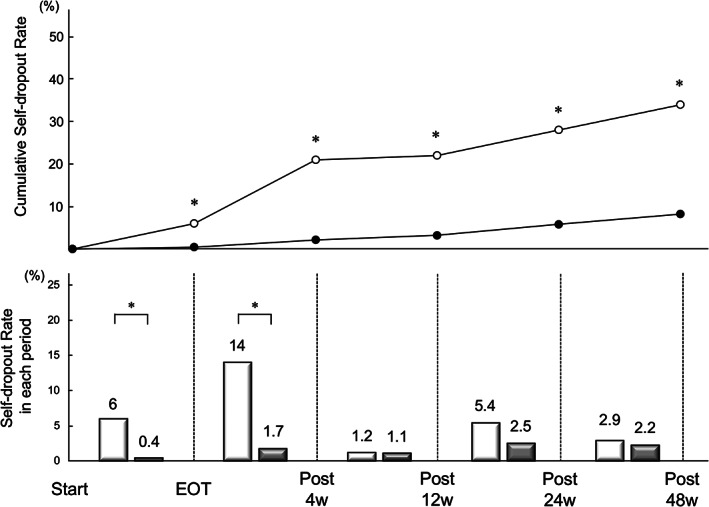
Dropout rate in the past‐IDU (injection drug use) group and in the non‐IDU group. Top, cumulative rate of dropout. The rate was significantly higher at every point in the past‐IDU group. Bottom, dropout rate during five separate periods. The rate was significantly higher in the past‐IDU group between start and EOT and between EOT and after 4 weeks. *P < 0.01, EOT, end of the treatment. 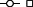, Past IDU; 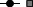, Non‐IDU

After 12 weeks, 44 patients had dropped out. The cumulative dropout rates were 6% (5/78), 21% (16/78), 22% (17/78), 28% (21/74), and 34% (23/68) at EOT and after 4, 12, 24, and 48 weeks in the past‐IDU group and 0.4% (3/726), 2.1% (15/726), 3.2% (23/726), 5.8% (41/713), and 8.2% (56/686) in the non‐IDU group (*P* < 0.01), respectively (Fig. 5). The dropout rate was significantly higher in the past‐IDU group in the period from the start to after 4 weeks (*P* < 0.01).

## Discussion

This is the first report on DAA therapy for patients with past‐IDU in Japan. In our cohort, 9.7% of DAA treatment‐naïve patients had a history of IDU. These patients had common characteristics at baseline compared to the non‐IDU group, consisting of younger patients with a male predominance. GT2‐infected patients were predominant in the past‐IDU group. Some of our findings are not consistent with those from other countries. In Australia and the Czech Republic, GT3 patients were predominant in PWID.^13^, ^14^ Previous studies in Japan have reported a high prevalence of GT2 in PWID.^15^, ^16^ It was speculated that a community‐specific HCV genotype had spread among PWID. In our study, the number of patients with advanced hepatic cirrhosis or HCC was lower in the past‐IDU group. Previous studies have reported that 22–30% of PWID with HCV have severe hepatic fibrosis, including cirrhosis.^17^, ^18^ In two studies, age, coinfection with human immunodeficiency virus (HIV), and obesity were associated with advanced hepatic fibrosis in PWID infected with HCV.^17^, ^18^ Although the rate of coinfection with HIV was not available for our cohort, the past‐IDU group was younger and less obese than the non‐IDU group. In another study, people with HCV and HIV had liver fibrosis stages similar to those without HIV who were nearly a decade older.^19^ We speculate that the stage of liver disease depends on the duration of HCV infection. Another common characteristic in patients with past‐IDU was social background. Overall, 63% patients with past IDU received public welfare assistance. In a previous study, 16% (145/915) of HCV‐infected patients with recent or past IDU were homeless, and 42% of them did not have an income.^20^ These results confirm that financial support is necessary for HCV‐infected PWID to improve their liver disease.

The selected 12‐week regimen of DAA achieved a high SVR rate for DAA‐naïve past‐IDU and non‐IDU patients. Our results are consistent with previous reports from other countries.^21^, ^22^ A systematic review reported a 5‐year HCV recurrence rate of 10.67%, driven mainly by reinfection in IDUs or prisoners.^7^ To eliminate HCV in PWID, long‐term follow‐up after DAA therapy is necessary. In our study, there was no HCV recurrence in past‐IDU patients who were followed up. However, the cumulative rate of dropout from the aftercare program was high in that group, especially up to 4 weeks after treatment. Previous studies have reported dropout rates of 29–35%.^21^, ^23^ We speculate that our dropouts may have been due to poor education in HCV therapy, lack of money, and the reinjection of drugs (two patients were incarcerated due to having illegal drugs; data not shown). Even when SVR is achieved, HCV reinfection can occur by injection with HCV‐contaminated needles. Meta‐analysis clearly showed that HCV reinfection risk after DAA treatment was higher in patients with recent IDU compared to those receiving opioid agonist therapy (OAT).^24^ We could not dismiss the possibility of HCV reinfection among dropout patients. A recent randomized control study showed that the intensive interventions resulted in greater adherence and higher SVR rate than self‐administered treatment in PWIDs who were treated with DAA.^25^ However, these findings were limited in PWIDs receiving opioid agonist therapy. It is necessary for past‐IDU patients to establish another support system.

There were several limitations to this study. First, this was a retrospective observational study conducted in one city of Japan. Moreover, the data were based on self‐assessment information in clinical records, not on direct questioning of patients. In addition, unlike previous studies on PWID, there were no patients who currently used injected drugs in this study. Furthermore, there were little available data on coinfection with HIV. However, no patients showed symptoms of HIV, and none of them were taking anti‐HIV drugs. Anti‐HIV antibodies were negative in all 17 past‐IDU patients who were tested (data not shown).

Taken together, our results indicate that DAAs had a remarkable anti‐HCV effect on patients with past IDU who continued in an aftercare program. To eliminate HCV in Japan and to inhibit progression of liver disease in PWID, it is necessary to understand the characteristics of PWID and to establish a support system from screening to aftercare for PWID.
